# Corporate governance and shareholders’ confidence in cooperative corporations: a systematic literature review

**DOI:** 10.12688/f1000research.73317.1

**Published:** 2022-02-03

**Authors:** Arasu Thangaveloo, Magiswary Dorasamy, Abdul Aziz Bin Ahmad, Siva Barathi Marimuthu, Jayamalathi Jayabalan

**Affiliations:** 1 VNK Bersatu Resources Sdn Bhd, Klang, Selangor, 41200, Malaysia; 2 Faculty of Management, Multimedia University, Cyberjaya, Selangor, 63100, Malaysia; 3 University of New England, Armidale, NSW, 2350, Australia; 4 Universiti Tunku Abdul Rahman, Kajang, Selangor, 43000, Malaysia

**Keywords:** Cooperative corporations, corporate governance, shareholders, B40, Malaysia, confidence, agency theory

## Abstract

**Background**: The confidence of Bottom 40 (B40) shareholders is crucial for cooperative corporation’s sustenance within wider corporate governance. An in-depth study on cooperatives is needed, as they play a crucial role in the Malaysian economic system and contribute greatly to the country’s social development. However, in the current landscape, confidence among shareholders is at stake. This study aims to identify the research gap into corporate governance for cooperative corporations in relation to B40 shareholder confidence, as well as identify current study challenges and develop a conceptual framework for future research.

**Methods**: We conducted a systematic literature review, with the use of agency theory to assess shareholders’ confidence. Emerald, ProQuest, InderScience, Scopus and Science Direct were the online databases used in this study to search five keyword phrases: corporate governance, confidence, cooperative, agency theory and Bottom 40% (B40) household. Tranfield’s five stages were used to conduct the systematic review.

**Results:** Only 5 of the 324 studies assess shareholders’ confidence in cooperatives, as well as one paper on B40 and two papers on agency theory. Our review presents three major findings. First, research in the context of B40 shareholder’s confidence in cooperative corporations is limited. Second, the challenges related to shareholders’ confidence in B40 are major issues in the context. Third, research on agency theory in the context of shareholders’ confidence within cooperative corporations and corporate governance is still scant.

**Conclusions:** This review urges the research community to conduct more studies based on the highlighted research gaps.

## Introduction

Malaysia’s cooperative movement began in the early twentieth century. A cooperative corporation is an organisation whose purpose is to enhance its members' economic value in line with cooperative goals. Consumer cooperatives, labour cooperatives, finance or banking cooperatives, and community cooperatives are only a few examples of cooperatives from various industries. Cooperative corporations play a crucial part in the Malaysian economic system and contribute greatly to the country’s social development. Their contributions are valued at the same level as those made by other businesses, in terms of the value-added to the national economy. ^1^ This study considers the corporate governance of cooperative corporations and its relation to shareholders’ confidence. Corporate governance is a system of internal rules, procedures and employees that meets shareholders and other stakeholders' requirements via the management and control of management operations with strong business skills, objectivity, responsibility and integrity.

A lack of confidence in the ability of the cooperative management, can in turn, could trigger a financial crisis within the corporation. Thus, the failure of a cooperative could become inevitable, which may eventually damage shareholders willingness to provide future investment. ^2^ The effect of shareholders’ views is even more critical in the case of cooperative corporations, because these companies often have political influence. Politically linked companies can possess bad corporate governance practices and higher-level corporate issues because they depend heavily on the federal government. ^3^ Thus, studying corporate governance’s impact on the confidence levels of shareholders in cooperative corporations is essential. Serious consequences may arise if it affects the confidence of Bottom 40 (B40) household shareholders. B40 refers to the bottom 40% in Malaysian household income; categorized as low-income householders earning below 4850 Malaysian Ringgit (RM) per month (approximately USD$1158). Various cooperatives corporation offered a share to the B40 employees or B40 members as one of the cooperatives benefits. Such cooperatives in Malaysia include the Armed Forces Cooperative, National Land Finance Co-operative Society (NLFCs), Koperasi Permodalan Felda Berhad and many more. Factors such as transparency, trustworthiness, integrity and good governance, are the key factors in creating shareholder confidence, and ensuring cooperative organisations remain competitive.

### Corporate governance

Malaysia’s awareness of the importance of corporate governance began after the 1997 financial crisis, which hit many Southeast Asian countries, resulting in a tremendous decline in share prices and currency value. ^4^ One major cause of the situation was the absence of confidence and honesty, as well as poor corporate governance within many organisations. ^5^^–^^7^ One of the country's oldest cooperatives, Malay Officers Cooperative Credit and Investment Society (MOCCIS), was declared bankrupt in 2008. The government also seized the accounts of three cooperatives in December 2008, after it was discovered that they had misled over 5,000 members into investing RM80 million in unlawful get-rich-quick schemes. Koperasi Taqwa Malaysia Berhad (Kotaqwa), Koperasi Ushahawan Malaysia Berhad (KUMB), and Koperasi Birr Berhad (Birr) were the three cooperatives (Consumers’Association of Penang). Literature has documented evidence that a reliable corporate governance framework can have a significant impact in improving strong efficiency along with emerging economic situations. ^5^^–^^7^

### Problems with cooperative corporation shareholders

For most scholars, researching the confidence of shareholders in a cooperative organisation is a challenge because of limited literature linking confidence to cooperative corporation. Therefore, in this study, we seek to describe the notion of shareholders’ confidence, in order to understand how it related to the cooperative corporation’s effectiveness. Agency theory may help to shed some light on the existing body of knowledge, to address the problems within this context.

### Agency theory and cooperative corporations

According to agency theory, businesses operate as agents for their shareholders. That is, shareholders engage in corporate ownership and pledge their assets to the management of the corporation's directors and officers. The relationship between cooperative governance and shareholder confidence is examined using the agency theory. ^8^ Agency theory is a relevant theory in this context that influences Malaysia’s corporate governance structures. This study’s analysis provides valuable information on how business management and shareholder confidence.

The research questions for this study are as follows:

1. Is there a research gap in B40 shareholders’ confidence in the cooperation corporation sector?
2. What are the challenges related to shareholders’ confidence in B40?
3. What will be the conceptual framework for B40 shareholders’ confidence in the cooperation corporation sector?
  The objectives of this proposal are as follows:
1. To identify if there is a research gap in B40 shareholders’ confidence in the cooperation corporation sector.
2. To understand the challenges related to shareholders’ confidence in B40.
3. To propose a conceptual framework for B40 shareholders’ confidence in cooperation corporation sector.

Two primary reasons for conducting a systematic literature review are as follows; firstly it enables researchers to locate and systematise existing literature on a specific research topic and determine the level of interest that scholars have shown in investigating a particular topic. Secondly, it adds to our understanding of the types of relationships studied in the context. This study aims to understand and evaluate the B40 shareholders’ confidence in cooperative corporations, concerning risk, expected return and confidence in future investment possibilities. This study is an initiative to identify the major challenges involved in corporate governance practices in cooperative firms.

## Methods

### Ethics

This study was approved by the Research Ethical Committee of Multimedia University, Malaysia (EA2712021).

This study is designed to present a literature review, research gap analysis, conceptual framework and insights gained on cooperative corporations and their relation to B40 shareholder’s confidence. The literature review is based on the five stages of systemic review proposed by Tranfield *et al*. ^9^

**Figure 1.**
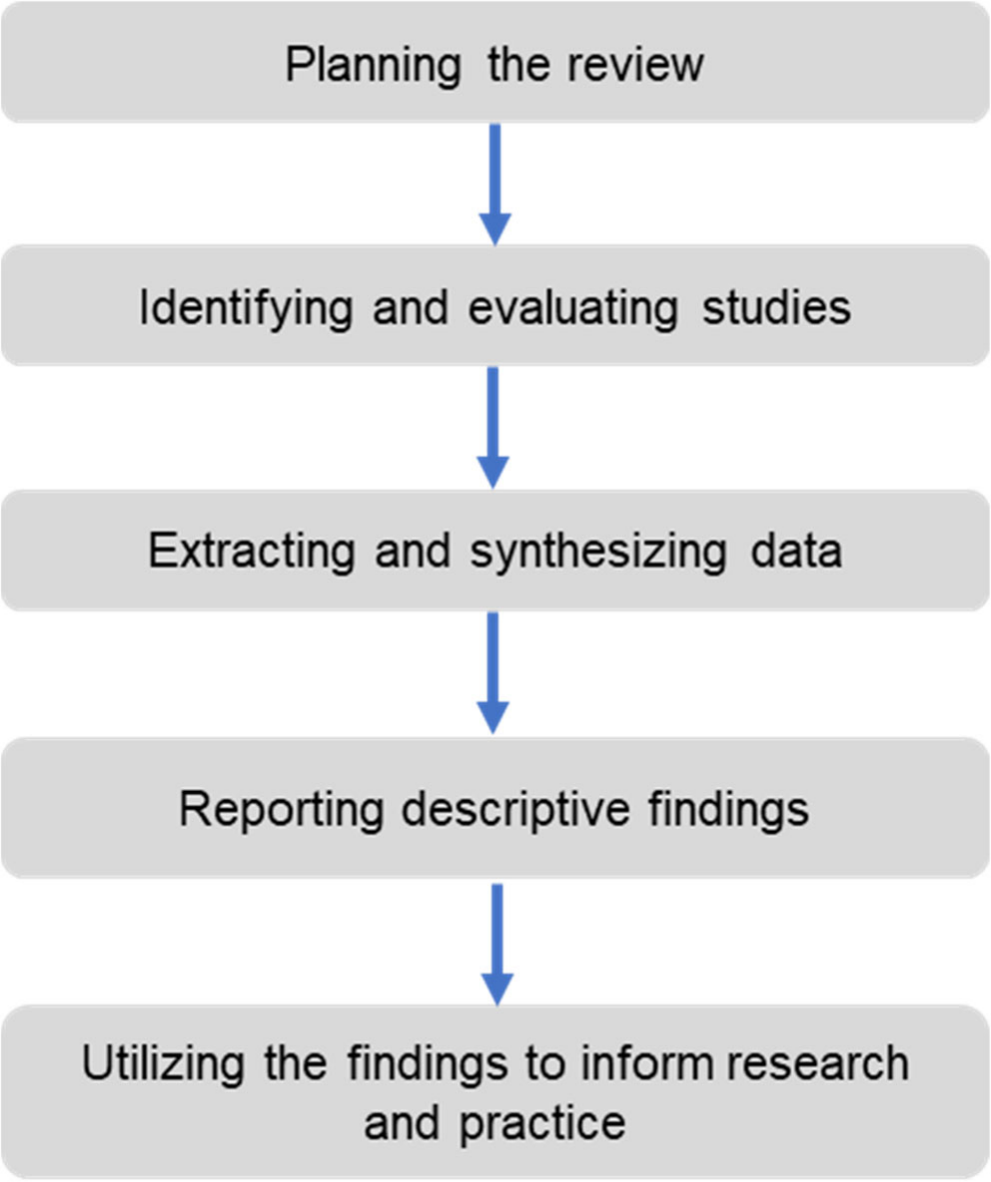
Five stages of systematic review.

Figure 1 below depicts the systematic flowchart of the review methodology.

### Identifying and evaluating studies

Articles investigating the confidence components in corporate governance within the context of cooperative corporations, do not specifically discuss the confidence of shareholders particularly in cooperative corporation. This study found that the Malaysian Code on Corporate Governance (MCCG) did not apply to cooperative corporation investors, in terms of there being no explicit regulations for how and when their practices may be improved. Effective corporate governance structure improves B40 shareholders confidence in terms of transparency, trustworthiness, integrity and good governance. As a result, we opted to check the number of publications for the specified keywords as the first step. The keywords range from broad themes (corporate governance) to more targeted terms (corporate governance/confidence/cooperative/B40).

*Criteria of inclusion and exclusion*

**Figure 2.**
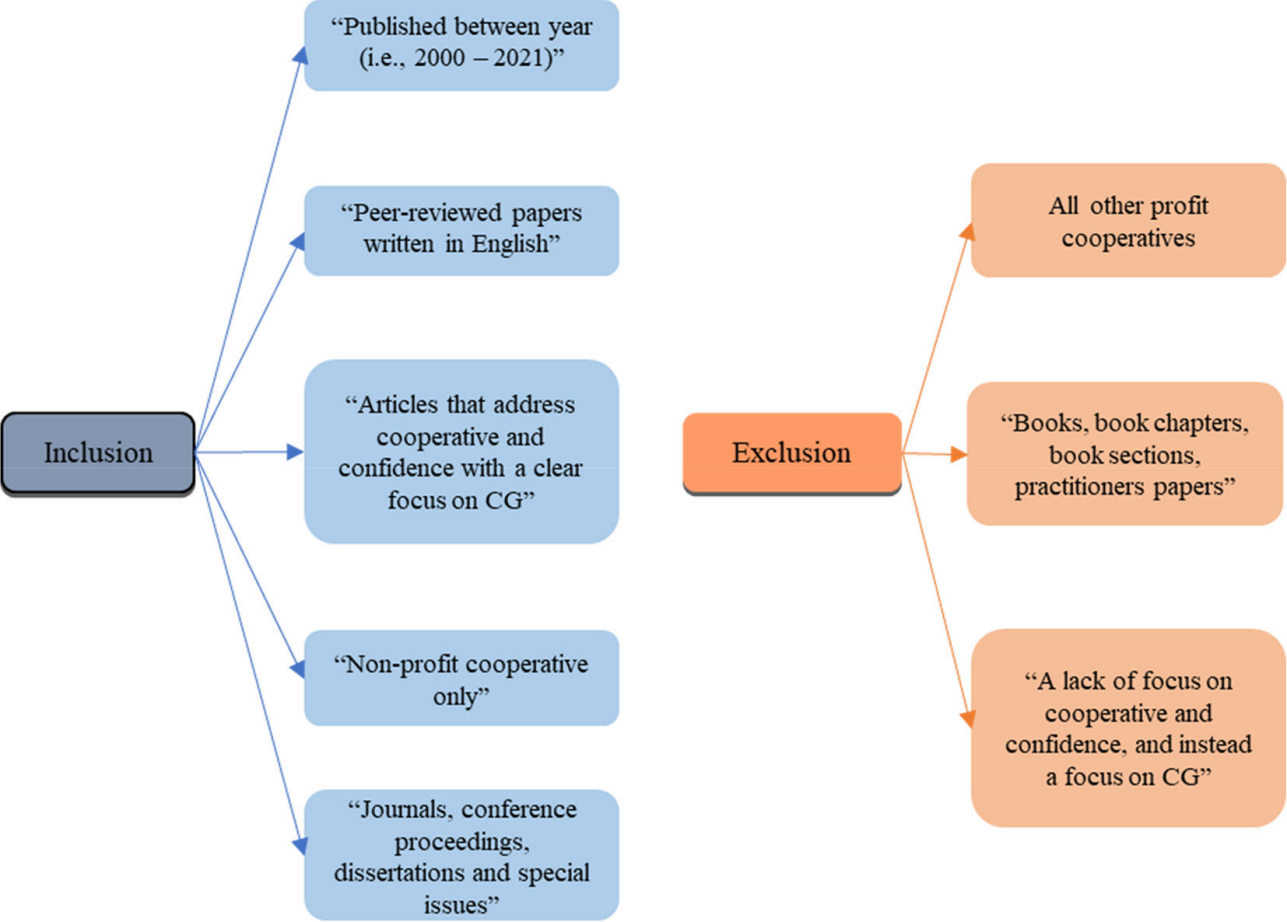
Inclusion and exclusion criteria.

In order to conduct the search for this study, the following exclusion and inclusion criteria was used, detailed in Figure 2 also. Papers were selected if they were published between year 2000 and 2021, had been peer-reviewed, written in English, and if they addressed non-profit cooperative corporations and corporate governance. Journals, conference proceedings, dissertations and special issues were included in the inclusion criteria. Five major databases that were used to conduct the searches; Emerald, Scopus, Science direct, Inderscience and ProQuest.

*Keywords*

In order to review the literature comprehensively, we followed Tranfield *et al*.,’s systematic literature review methods, and used Emerald, ProQuest, InderScience, Scopus and Science Direct to search the following keywords: (1) corporate governance, (2) confidence, (3) cooperative, (4) agency theory and (5) B40. ^9^ The phrases searched included: ‘corporate governance’, ‘corporate governance and investors/shareholders confidence’, ‘corporate governance and investors/shareholders confidence and cooperative’, ‘corporate governance and investors/shareholders confidence and B40’ and ‘corporate governance and investors/shareholders confidence and cooperative and B40’. The search began with more general searches using the terms ‘corporate governance’ and/or ‘confidence.’ The searches were conducted between 1st June 2021 and 15th June 2021.

*Search strategy*

The search strategy was based on the keyword search, in order to investigate papers in the five major online databases, including Emerald, ProQuest, InderScience, Scopus and Science Direct. This selection was based on these databases’ widespread use and high level of academic merit. ^11^ Furthermore, these databases include a large number of periodicals devoted to business and cooperatives. The keywords selected were combined into five possible combinations, as shown in Table 1. The search on ‘corporate governance’ alone initially produced 77,864 publications. When searched with ‘corporate governance and investors/shareholders confidence’, the number dropped to 2,304. When we used the keyword combinations of ‘corporate governance and investors/shareholders confidence and cooperative,’ the number was 324. Subsequently, we found only one publication when we searched for ‘corporate governance and investors/shareholders confidence and B40’. The same was also true when we searched for ‘corporate governance and investors/shareholders confidence and cooperative and B40’; only one publication was found.

**Table 1.** Summary of keyword results.

No.	Online database	Corporate governance	Corporate governance AND Investors’ confidence OR Shareholders confidence	Corporate governance AND Investor/shareholders confidence AND Cooperative	Corporate governance AND Investor/shareholders confidence AND B40	Corporate governance AND Investor/shareholders confidence AND Cooperative AND B40
1	Emerald	18000	898	65	0	0
2	ProQuest	16524	482	171	0	0
3	InderScience	3546	105	28	0	0
4	Scopus	21766	118	0	0	0
5	Science Direct	18028	701	60	1	1
	**Total:**	**77864**	**2304**	**324**	**1**	**1**

**Figure 3.**
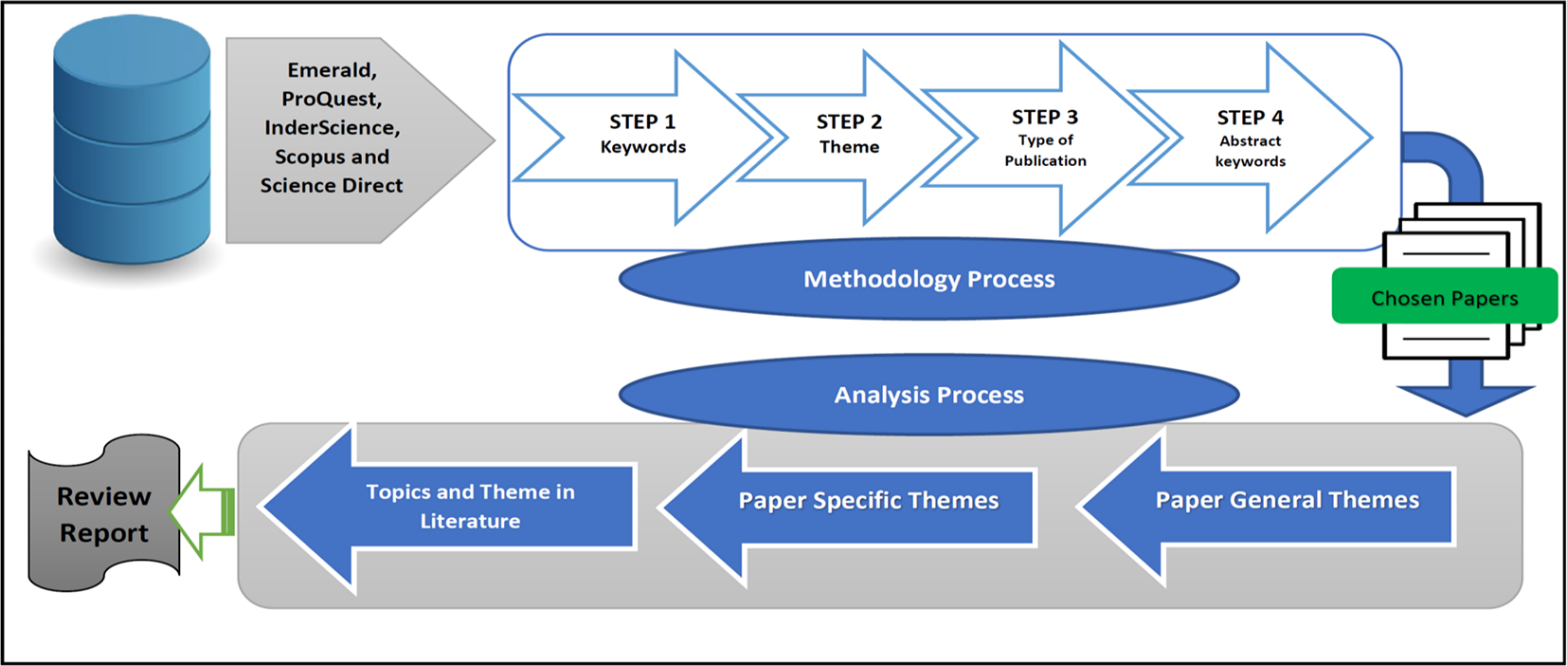
Extraction process.

We used the following technique to extract papers from the major web databases mentioned above, summarised in Figure 3. Only confidence in cooperative cooperation papers linked to the B40 group, were selected for further evaluation.

## Results

From the 324 papers found through searching for ‘corporate governance and investors/shareholders confidence and cooperative, we found five papers relevant to our investigation. Only 5 of the 324 studies assess shareholders’ confidence in cooperatives, as well as one paper on B40 and two papers on agency theory. The findings are summarised in Table 2.

**Table 2.** Summary of core papers.

Author(s)	Theory	Respondents	Research method	Findings	Challenges/limitations
^13^Shahid & Abbas (2019)	Market timing theory, Catering theory	Non-financial listed firms	Mixed methods	Confidence financial investment level is higher in the firms with good company governance practices.	Nil
^4^Gould, Melecky & Panterov (2016)	Nil	Ten-year averages of data for more than 100 countries over 1970–2014	Quantitative	Significant effect of income convergence, as growth (overall and for the poorest 40%) is slower in countries with higher initial income levels.	Causal link from finance to growth is notoriously difficult to prove.
^15^Zekos (2003)	Neoclassical theory	SME & MNE	Qualitative	Organizational learning capacity (OLC) is significantly related to customer orientation within strategic sourcing units.	Market definition is made more complex; market data is often hard to find.
^16^Norman & Norman (2016)	Agency theory, stewardship theory, economic theory	CEO’s	Qualitative	More than 75% of chief executives are said to have “golden parachute” regardless of their performance in failed companies.	The paper was largely undertaken by the analysis of secondary data sources.
^17^Mardini & Lahyani (2020)	Agency theory, impression management theory	Non-financial SPF-120 French listed firms	Quantitative	FP, measured using both market (Tobin's q) and accounting (return on equity and return on assets) indicators.	The scoring method used intellectual capital disclosures (ICDs) relating to human capital (HC), structural capital (SC) and relational capital (RC).

Although most of these studies have focused on the direct impact of confidence in cooperative corporations, some authors are interested in investigating the confidence effect that significant shareholders can exert. Only 5 of the 324 studies, displayed in Table 2, discuss shareholders’ confidence in cooperatives, including one paper on B40, two papers on agency theory and none on non-profit cooperatives. The results of the analysis are presented in Table 3.

**Table 3.** Summary of theories focused.

Theory	No of papers	Authors (Year)
Agency theory	2	^16^Norman and Norman (2106), ^17^Mardini and Lahyani (2020)
Market timing theory	1	^13^Shahid, and Abbas (2019)
Catering theory	1	^13^Shahid, and Abbas (2019)
Neoclassical theory	1	^15^Zekos (2003)
Stewardship theory	1	^16^Norman and Norman (2016)
Economic theory	1	^16^Norman and Norman (2016)
Impression management theory	1	^17^Mardini and Lahyani (2020)

Table 3 lists two papers with agency theory and six other theories, which were used in the studies based on different context.

## Discussion

There are several gaps identified in this study. There are limited studies on corporate governance related to B40 shareholders confidence in the context of cooperative corporations. More research and case studies are crucial for cooperative corporations to understand and adopt any possible recommendations; the implication of which would be to encourage B40 shareholders to invest in such corporations.

A limitation of our study is the number of keywords selected; it would have been possible to obtain more papers in our search if the keywords were expanded to broader fields of study, not so specific in nature, such as B40. This could possibly lead to publication biases.

*Research gap in B40 shareholders’ confidence in the cooperation corporation sector*

Very few papers discuss the importance of the B40 shareholders’ confidence in cooperative corporations. Filling the research gap is vital to building more knowledge of B40 shareholders in investment portfolios; which will help cooperative corporations build confidence in shareholders as a critical feature. The data are shown in Tables 4 and 5.

**Table 4.** Plotting of papers on shareholder confidence in cooperation corporations, B40, agency theory and non-profit cooperatives.

Author(s)	Shareholder confidence in corporative cooperation	B40	Agency theory	Non-profit cooperative
^13^Shahid & Abbas (2019)	**√**			
^14^Gould, Melecky & Panterov (2016)	**√**	**√**		
^15^Zekos (2003)	**√**			
^16^Norman & Norman (2016)	**√**		**√**	
^17^Mardini & Lahyani (2020)	**√**		**√**	

**Table 5.** Summary of papers on shareholder confidence in cooperation corporations, B40, agency theory, non-profit cooperative.

Research gaps	No of papers	Authors (Year)	Key takeaways/points to discuss
Shareholder confidence in cooperative corporations	5	^13^Shahid, and Abbas (2019), ^14^Gould, Melecky & Panterov (2016), ^15^Zekos (2003), ^16^Norman & Norman (2016), ^17^Mardini & Lahyani (2020)	Overall, the articles did not highlight the shareholders confidence for future investment so there is a gap to be analysed.
B40	1	^14^Gould, Melecky & Panterov (2016)	The articles did not discuss briefly on B40 investors. Key consideration in adopting B40 investors into the study.
Agency theory	2	^16^Norman &Norman (2016), ^17^Mardini & Lahyani (2020)	The agency theory does not test some relationship between B40 shareholders confidence, CC and CG.
Non-profit cooperative	Nil	Nil	Zero publication on B40 Shareholders Confidence and non-profit cooperative.

*Challenges related to shareholders’ confidence in B40*

The main challenges with shareholders confidence in cooperative corporation is that it doesn't stand alone. The challenges related to shareholders’ confidence, in general, can be grouped into four categories, including external, internal, government related and population size. The external challenges include corporate tax and social behaviour of shareholders. Restrictive government policies and the high populations in B40 groups are another challenge related to shareholders confidence in B40s. However, these challenges do not sufficiently address the issues faced by B40 shareholders, which is one of the research gaps this study has identified. Furthermore, B40 shareholders are generally illiterate and need more knowledge about investment portfolio.

*Conceptual framework for B40 shareholders’ confidence in the cooperation corporation sector*

Agency theory has not been tested for B40 shareholders, particularly to address shareholders’ confidence in cooperation corporations. Shareholder’s confidence is a missing piece within the agency theory; especially in cooperative corporations. Protecting the interests of B40 shareholders who invest in the company and have expectations about the business and their investments, are critical. ^12^ Thus, we propose the following conceptual framework for further studies.

**Figure 4.**
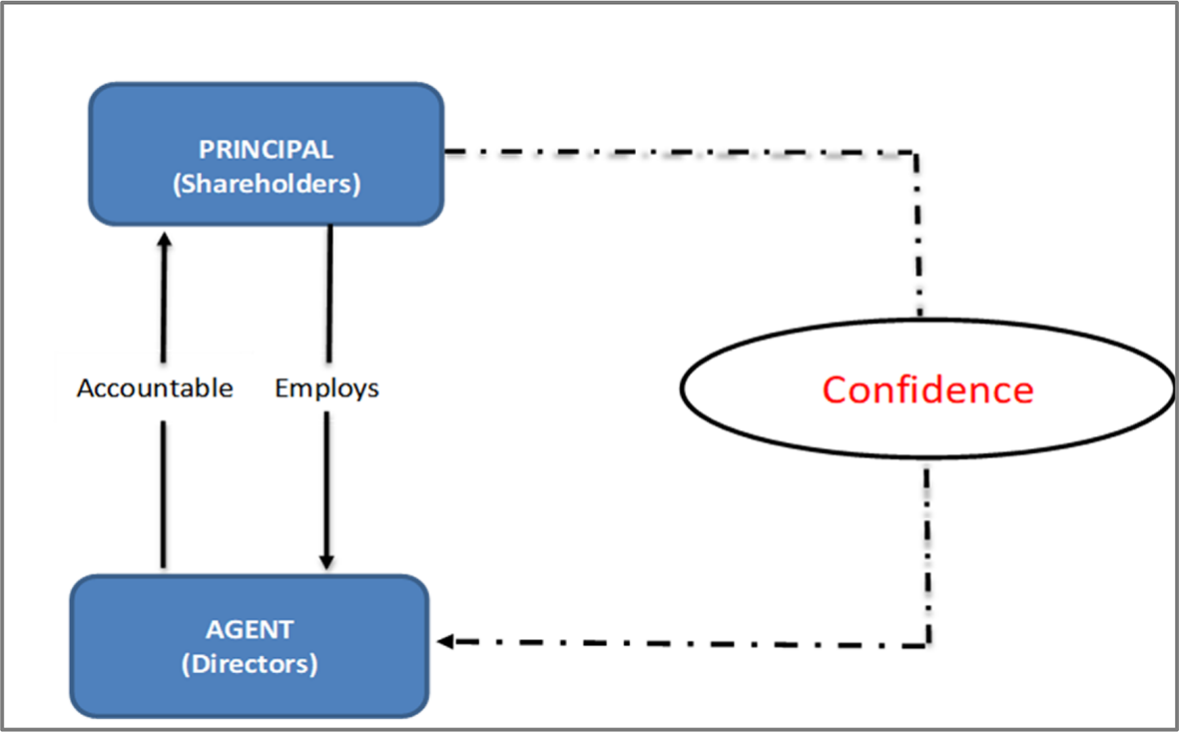
Conceptual framework.

## Future recommendations

Researchers looking into the association between B40 shareholder confidence, corporate governance and cooperative corporations could use the primary findings of this publication, to generate ideas for future research. Two primary research themes could be considered in the future and we hope the academic community conduct more research on the following:

### Corporate governance in cooperative corporation for M40 shareholders

Future studies should focus more on the Middle 40 (M40) shareholders, because they are the main contributors to the national GDP. M40 households make up 40% of Malaysia’s population, includes mostly wage earners, in public and private sectors, earning between RM4850-10959 per month.

## Conclusion

The purpose of this study is to update corporate governance and cooperative corporations research communities about research gaps, namely in the areas of shareholder confidence, particularly among B40 shareholders. No unique confidence metric exists today, nor a case study explicitly dedicated to confidence. In this study, we used a five-stage technique to review papers based on a thorough examination of the literature in a particular field. According to our in-depth analysis of these papers, there are three primary areas which have room for research. Firstly, there is a pressing need for researchers to standardise the terminologies used the discuss corporate governance, cooperative corporations and shareholder confidence. Secondly, more research is needed to determine whether corporate governance, cooperative corporations, shareholder’s confidence and the B40 groupto form the basis for more effective policy formulation and the basis for guiding the next generation of effective investment. In this context, the extent of the commonalities between shareholder confidence and B40 could be investigated further. Finally, further empirical research is needed to understand the elements that influence B40 shareholders’ confidence in the context of corporate governance and cooperative corporations.

## Data availability

### Underlying data

All data underlying the results are available as part of the article and no additional source data are required.

### Reporting guidelines

Figshare: Prisma checklist and data flow diagram doi.org/10.6084/m9.figshare.16822225

This project contains the following extended data:

• Data file 1. (Checklist, DOCX format)
• Data file 2. (Flow diagram, PDF format)
