# Supplementary material for: Corporate governance and shareholders’ confidence in cooperatives: a systematic literature review
Source: F1000Res. 2024 Mar 4;11:144. Originally published 2022 Feb 3. [Version 3] doi: 10.12688/f1000research.73317.3 (PMC10905109; doi:10.12688/f1000research.73317.3)
Supplement: Peer review history

# Reviewer report: Reviewer response for version 2

**DOI:** 10.5256/f1000research.122224.r127021

**Published:** 2022-03-25

**Authors:** Ravichandran K. Subramaniam

**Affiliations:** 1 School of Business, Monash University Malaysia, Subang Jaya, Malaysia

**Response to author's comment:**

 Author - *We have removed the non-profit cooperative component and used the cooperatives category in general.*

 Reviewer - Satisfactorily done

 Author - *We have now emphasized the scarcity of the B40 related research.*

 Reviewer - Satisfactorily done

 Author - *Rationale for low research in B40 Shareholders and how to address it in the future is now added.*

 Reviewer - Satisfactorily done

 Author - *Changed 'Cooperative corporations' to 'Cooperatives'.*

 Reviewer - Satisfactorily done

 Author - *Elaborated on corporate governance framework. Figure 2 was revised*.

 Reviewer - Satisfactorily done

Are the rationale for, and objectives of, the Systematic Review clearly stated?

Yes

Is the statistical analysis and its interpretation appropriate?

Yes

Are sufficient details of the methods and analysis provided to allow replication by others?

Yes

Are the conclusions drawn adequately supported by the results presented in the review?

Partly

Reviewer Expertise:

I have done Corporate governance-related papers and as such I am fine to review this paper

I confirm that I have read this submission and believe that I have an appropriate level of expertise to confirm that it is of an acceptable scientific standard.

# Reviewer report: Reviewer response for version 1

**DOI:** 10.5256/f1000research.76961.r122503

**Published:** 2024-02-07

**Authors:** Rajeswari R

**Affiliations:** 1 Jai Bharath Arts and Science College, Perumbavoor, India

The paper has been well drafted with a precise introduction, research strategy, results drawn, and the conclusion derived. The research questions and objectives set for the study clearly suit the purpose of the study. However, the study can be improved to make it error free.

 Main issues:

1. Since the word “Cooperative Cooperation” may be new to many readers, it would be appropriate to clarify if the term “Cooperations” and “Cooperative Cooperative” have different meanings.
2. Can mention more about the bottom 40 shareholders and the ways in which it can change the economic development of Malaysia.
3. Even though, the authors have stated about the scarcity of resources for collection of data on such a topic, authors should emphasise more on this matter, collect the data, analyse it and take the required information for the study.
4. Misspelled “cooperations” as “cooperatives” in many areas of the study.
5. It will be better if the authors have more suggestions on the future of such a topic and to influence more people to conduct study in this subject.

Are the rationale for, and objectives of, the Systematic Review clearly stated?

Yes

Is the statistical analysis and its interpretation appropriate?

Yes

Are sufficient details of the methods and analysis provided to allow replication by others?

Yes

Are the conclusions drawn adequately supported by the results presented in the review?

Yes

Reviewer Expertise:

Marketing, International Business, and Logistics and Supply Chain Management.

I confirm that I have read this submission and believe that I have an appropriate level of expertise to confirm that it is of an acceptable scientific standard.

# Reviewer report: Reviewer response for version 1

**DOI:** 10.5256/f1000research.76961.r122501

**Published:** 2022-02-21

**Authors:** Ravichandran K. Subramaniam

**Affiliations:** 1 School of Business, Monash University Malaysia, Subang Jaya, Malaysia

This paper has been well written with a clear introduction, comprehensive literature, search strategy, and overall results. The theory is sound and backed up with research questions and objectives. However, here are my pointers for the authors to improve the article in order for it to be of indexable quality:

**Main Issues:**

1. As there were no info/data available on 'Non-profit Cooperatives', then it is better to just stick to a study on 'Cooperatives' as otherwise, we will be misrepresenting the research.
2. Based on the overall literature sourced, there has only been one study on B40 out of 77,864. So the authors need to emphasize the scarcity of this study instead of downplaying it by saying it is merely limited. This is a significant finding.
3. With regards to contribution the authors could mention the rationale for such low research and how this could be addressed in the future.
4. Using the words 'Cooperative corporations', is rather confusing. Suggest simplifying it to the word 'Cooperatives'.

**Other Issues:**

1. Need to have a clear definition of 'Cooperatives' to avoid ambiguity.
2. Suggest elaborating on the term 'corporate governance framework'.
3. Under the research questions and objectives portion, instead of 'Cooperatives', the authors misspelled it as 'cooperation' and this has been repetitive.
4. In Figure 2, indicated as 'remove non-profit Cooperatives' and exclude 'profit Cooperatives' to rectify appropriately.
5. Very few papers discuss the importance of the B40 shareholders’ mentioned under the heading "Research gap in B40 shareholders’ confidence in the cooperative corporation sector" is not correct as there were only one (as per table 1).
6. Table 4 & 5 on the non-profit corporations, can be removed to maintain clarity and consistency. 

Are the rationale for, and objectives of, the Systematic Review clearly stated?

Yes

Is the statistical analysis and its interpretation appropriate?

Yes

Are sufficient details of the methods and analysis provided to allow replication by others?

Yes

Are the conclusions drawn adequately supported by the results presented in the review?

Partly

Reviewer Expertise:

I have done Corporate governance-related papers and as such I am fine to review this paper

I confirm that I have read this submission and believe that I have an appropriate level of expertise to confirm that it is of an acceptable scientific standard, however I have significant reservations, as outlined above.
